# Book Review: Embryogenesis Explained

**DOI:** 10.3389/fcell.2017.00079

**Published:** 2017-09-07

**Authors:** Alessandro Minelli

**Affiliations:** Biology, University of Padova Padova, Italy

**Keywords:** cell state spliiter, cytoskeleton, developmental disparity, differentiation, mechanotransduction, cellular, model species

In 1999, Richard Gordon, at that time Professor of Radiology at the University of Manitoba, Winnipeg, published a two-volume work under the ambitious title *The Hierarchical Genome and Differentiation Waves: Novel Unification of Development, Genetics and Evolution*. Eighteen years later, Gordon and his wife and former colleague Natalie present the *differentiation waves theory* to a broader public. Writing this book was arguably a reaction to the scanty attention hitherto paid to this theory. The new book, however, is much more than a mean to expose it anew. It is also autobiography and, additionally, a lavishly illustrated though unsystematic summary of basic issues in embryology, developmental genetics, cell biology and epigenetics.

The differentiation waves theory shifts the focus away from the genes, to look instead at the cytoskeleton as the *primum movens* of the process: this means leaving molecular genetics in the background, concentrating instead on mechanics. Starting point are Richard Gordon's observations on the embryo of a model vertebrate species, the axolotl. The backbone of the theory is articulated as follows:

1. at early gastrulation, in each ectodermal cell there is a complex of cytoskeletal elements composed of a ring of microfilaments, a mat of microtubules at the apical surface and parallel to it, and a ring of intermediate filaments. Curiously, but not mentioned in the book, this is quite similar to the apical complex of the Apicomplexa, a group of protozoans that includes *Plasmodium*, except for the absence of two secretory organelles called the rhoptries
2. because of its peculiar organization, this cytoskeletal complex is able to direct cell differentiation, hence the name of cell state splitter
3. contraction vs. expansion of the microfilament ring, accompanied by expansion vs. contraction of the microtubule mat, determines the two opposite stable states that the cell state splitter can take
4. the change of state in a cell's state splitter propagates to the adjacent cells, determining a wave that moves across the embryo
5. this wave acts as a signal that induces a change in genes expression
6. this change is limited to a “differon,” a set of genes whose expression is specific of a particular cell type

When Richard Gordon first introduced the concept of cell state splitter more than 20 years ago, mechanotransduction was not yet well established as a component of developmental processes as it is today. However, experimental evidence in favor of embryonic differentiation waves based on the putative cell state splitter has remained circumstantial.

If we accept that the cell splitter model and the differentiation waves act as a mechanism for cell differentiation in the axolotl, this does not say anything about how general the mechanism can be. This is however crucial for the Authors, who regard universality as the single most important quality of a “right” theory (p. vii). Throughout this book, they often concede that the model applies less controversially to regulative embryos, in which the fate of individual cells is not determined by position or lineage, as in the axolotl, than to mosaic embryos, of which they take *Caenorhabditis elegans* as example. A more serious problem, however, is represented by those developmental systems in which there is no embryo at all, yet cell differentiation occurs, as in the budding process e.g., in hydra and in colonial ascidians such as *Botryllus*. In the latter, zooids with nearly identical architecture and with the same array of cell types are obtained either by budding or from fertilized eggs developing into embryos, larvae and eventually adult zooids. In this case at least, embryonic waves are not required to obtain cell differentiation.

Eventually, this book can provide beneficial stimuli through both its strong and its weak aspects. On the strong side, it contributes to shaking the persisting gene-centrism by focussing on the contribution of factors other than genes in controlling development. Whether this should be looked for in still poorly documented parts of the cytoskeleton, as the putative cell state splitter, remains doubtful to me. But the passionate pages of the Gordons' book cannot fail to stimulate research into cell components involved in mechanical rather than molecular games—another player being membranes—of importance in development.

A weak point, as mentioned, is the unwarranted trust in the heuristic value of concentrating on model species. It is time to take into account the disparity of developmental systems and of the multiple alternatives sometimes open to one and the same organism. The difficulties found in extrapolating from the axolotl to animals with mosaic embryos should act as a stimulus toward a broad and cautious comparative approach. On a broader level, reading this book invites rethinking the legitimacy of describing as embryos the earliest developmental stages of organisms that have acquired multicellularity independent from animals—e.g., brown algae and green plants, including those known as the Embryophyta (mosses, ferns, and seed plants). Terminology apart, although multiple developmental roles have been recently described for the cytoskeleton in the flowering plants, there is no hope to find their cells, solidly encased within a stiff wall, to be affected by cell differentiation waves.

Innovative and provocative as a model of differentiation based on cytoskeletal structure and dynamics can be, the Gordons' approach is conservative in other respects—again, a number of points that deserve fresh revisitation. To list only three of these: are cell types unequivocally distinct? are these obtained by a sequence of choices along a programmed hierarchical tree, even not necessarily binary as it should be, if this depends on a bistable cell state splitter? are the differential transcriptomes corresponding to different cell states, or different developmental stages, in some way embodied in the genome's architecture? To be sure, in the genome of a sea urchin there are not two compartments, one for “larval genes,” the other for “adult genes”; however, is there anything responsible, at the genomic level, for the broad and independent conservation of the larval vs. adult architecture in the evolution of animals groups undergoing dramatic metamorphoses?

## Author contributions

The author confirms being the sole contributor of this work and approved it for publication.

### Conflict of interest statement

The author declares that the research was conducted in the absence of any commercial or financial relationships that could be construed as a potential conflict of interest.

